# A Bayesian Network Meta-Analysis of First-Line Treatments for Non-Small Cell Lung Cancer with High Programmed Death Ligand-1 Expression

**DOI:** 10.3390/jcm11061492

**Published:** 2022-03-09

**Authors:** Jung Han Kim, Soo Young Jeong, Jae-Jun Lee, Sung Taek Park, Hyeong Su Kim

**Affiliations:** 1 Division of Hemato-Oncology, Department of Internal Medicine, Kangnam Sacred-Heart Hospital, Hallym University Medical Center, Hallym University College of Medicine, Seoul 07441, Korea; harricil@hallym.or.kr; 2 Department of Obstetrics and Gynecology, Kangnam Sacred-Heart Hospital, Hallym University Medical Center, Hallym University College of Medicine, Seoul 07441, Korea; sygy19@hallym.or.kr; 3 Institute of New Frontier Research Team, Hallym University, Chuncheon 24253, Korea; iloveu59@hallym.or.kr; 4 Departments of Anesthesiology and Pain Medicine, Chuncheon Sacred-Heart Hospital, Hallym University Medical Center, Hallym University College of Medicine, Chuncheon 24253, Korea

**Keywords:** non-small cell lung cancer, immune checkpoint inhibitor, immune evasion, Bayesian meta-analysis, review

## Abstract

We performed a Bayesian network meta-analysis (NMA) to suggest frontline treatments for advanced non-small cell lung cancer (NSCLC) showing high programmed death ligand-1 (PD-L1) expression. A total of 5237 patients from 22 studies were included. In terms of progression-free survival, immune checkpoint inhibitors (ICIs) plus bevacizumab plus chemotherapy had the highest surface under the cumulative ranking curve (SUCRA) value (98.1%), followed by ICI plus chemotherapy (82.9%). In terms of overall survival (OS), dual immunotherapy plus chemotherapy had the highest SUCRA value (79.1%), followed by ICI plus bevacizumab plus chemotherapy (73.4%). However, there was no significant difference in survival outcomes among treatment regimens combined with immunotherapy. Moreover, ICI plus chemotherapy failed to reveal a significant OS superiority to ICI monotherapy (hazard ratio = 0.978, 95% credible internal: 0.771–1.259). In conclusion, this NMA indicates that ICI plus chemotherapy with/without bevacizumab might to be the best options in terms of OS for advanced NSCLC with high PD-L1 expression. However, considering that there was no significant difference in survival outcomes among treatment regimens incorporating immunotherapy and that ICI plus chemotherapy failed to show significant survival benefits over ICI monotherapy, ICI monotherapy may be reasonable as first-line treatment for advanced NSCLC with high PD-L1 expression.

## 1. Introduction

Lung cancer is the leading cause of cancer-related death all over the world [1]. Nearly half of all patients with non-small cell lung cancer (NSCLC) are presented in the advanced or metastatic stages, which limits the treatment options. For a long time, platinum-based doublet chemotherapy was the first-line standard treatment for patients with advanced NSCLC without driver somatic mutations [2].

Recently, cancer immunotherapy has been established as a new treatment option for many solid tumor types, including advanced NSCLC [3,4]. Immune checkpoint inhibitors (ICIs) refer to monoclonal antibodies (mAbs) engineered to block co-inhibitory molecules such as CTLA-4, anti-programmed death-1 (PD-1), and PD-ligand 1 (PD-L1) and restore antitumor immunity [5,6]. Randomized trials have revealed that anti-PD-1 mAbs (pembrolizumab and nivolumab) and anti-PD-L1 mAb (atezolizumab) provide additional benefits in both overall survival (OS) and progression-free survival (PFS) for patients with previously treated advanced NSCLC, compared with chemotherapy [7,8,9,10,11]. ICIs are also recommended as a first-line treatment for advanced or metastatic NSCLC, either as monotherapy or in combination with chemotherapy or other targeted agents, based on histology, genetic alterations, and level of PD-L1 expression [12,13,14,15,16,17,18,19,20,21,22,23,24,25,26,27,28,29,30,31,32,33,34,35,36].

The level of PD-L1 expression is currently the best predictive biomarker for the efficacy of ICIs in advanced NSCLC, although its predictive power is limited, especially in the combination treatment with cytotoxic agents. Approximately 25–35% of advanced NSCLC cases are expected to test positive for PD-L1 in at least 50% of tumor cells by immunohistochemistry (IHC) [7,12,21]. ICI monotherapy (pembrolizumab, atezolizumab, and cemiplimab) has significantly improved survival outcomes (PFS or OS) compared with chemotherapy in the first-line treatment for advanced NSCLC with PD-L1 expression of at least 50% and without epidermal growth factor receptor (*EGFR*), anaplastic lymphoma kinase (*ALK*), or *ROS* oncogene 1 (*ROS1*) aberrations [12,21,24].

Currently, there are a variety of anti-cancer drugs available in the first-line treatment of advanced NSCLC, such as cytotoxic agents, targeted agents, and ICIs [3,4,5,6,7,8,9,10,11]. ICIs have transformed the paradigm of treatment for advanced NSCLC without *EGFR*, *ALK*, or *ROS1* aberrations [12,13,14,15,16,17,18,19,20,21,22,23,24,25,26,27,28,29,30,31,32,33,34,35,36]. However, randomized trials investigating the efficacy of ICIs as monotherapy or in combination with chemotherapy or other targeted agents are lacking for advanced NSCLC with high PD-L1 expression. Thus, there are needs to optimize first-line treatment options for patients with advanced NSCLC highly expressing PD-L1.

In the absence of head-to-head trials, a Bayesian network meta-analysis (NMA) can allow us to combine both direct and indirect evidence and compare several therapeutic regimens using a common comparator in the individual trials [37]. To give an overview of the current status of immunotherapy in advanced NSCSC and suggest optimal frontline treatments for patients with high PD-L1 expression, we performed a systematic literature review and NMA of randomized clinical trials.

## 2. Materials and Methods

### 2.1. Searching Strategy

We searched the Cochrane Central Register of Controlled Trials, PubMed, and EMBASE for articles that included the following search terms in their titles, abstracts, or keyword lists: ‘metastatic or advanced’, ‘non-small cell’, ‘lung’, ‘malignant or neoplasm or cancer or carcinoma’, ‘treatment’, ‘chemotherapy’, ‘immune checkpoint inhibitor or immunotherapy’ and ‘randomized or randomised’. All eligible studies were retrieved, and their bibliographies were checked for other relevant publications. We also scanned the reference lists of relevant articles and reviews. In addition, we used the ‘related articles’ features in PubMed to identify other potentially eligible articles. In the case of duplicate publications, the more recent paper was selected. Two independent reviewers examined the titles, abstracts, and full articles to determine the eligibility of the identified trials. Any disagreements were resolved through consensus or consultation with a third reviewer.

### 2.2. Selection Criteria

All potentially eligible studies identified using the search strategy were screened. Clinical trials that met the following criteria were reviewed for the NMA: (i) prospective randomized phase II or III trials for advanced NSCLC; (ii) trials comparing treatment regimens in the first-line setting; (iii) trials reporting the efficacy according to the level of PD-L1 expression or studies conducted for advanced NSCLC with greater than or equal to 50% PD-L1 expression.

### 2.3. Definition of High PD-L1 Expression

High PD-L1 expression is defined as a tumor proportion score (TPS) ≥ 50% or as either ≥50% of tumor cells (TC; TC3) or ≥10% of tumor-infiltrating immune cells (IC; IC3) [4].

### 2.4. Data Extraction

Two independent reviewers extracted the complete data from each included trial using a standardized data extraction form. Extracted data included the details of the trials (year of publication, treatments, number of patients, and histology) and outcome measures (PFS and OS). The risk of bias for each trial was assessed by the Cochrane risk of bias method. Discrepancies in data extraction were resolved through discussion.

### 2.5. Data Analysis

The primary outcomes intended to analyze were OS and PFS, which were reported as a hazard ratio (HR) and its 95% confidence intervals (CIs). A Bayesian NMA was conducted to evaluate the treatment effects by direct pairwise and indirect comparisons and to provide a hierarchical ranking for the treatments without direct comparisons between them. Considering the heterogeneity between included trials, a random-effects model was incorporated and an informative prior of a log-normal (−3.95, 1.342) distribution was set in the Bayesian framework [38].

The posterior distributions were obtained using Markov-chain Monte Carlo process with 5000 burn-ins and 50,000 iterations of four chains, which were thinned after every 10th simulation to reduce autocorrelation [39]. The convergence of the model was assessed by evaluating the trace plots and Gelman–Rubin diagnostics with a cut-off value of 1.05 [40]. The effect sizes of the Bayesian NMA were presented as the HR with 95% credible intervals (CrIs). To provide the rankings of each treatment, the surface under the cumulative ranking curve (SUCRA) values were calculated. A higher SUCRA value indicates a higher the likelihood that the treatment option would be in the top rank [40].

The statistical heterogeneity was evaluated using the statistic inconsistency index (*I*^2^). *I*^2^ values of <25%, 25–50%, and >50% indicate low, moderate, and high heterogeneity across randomized controlled trials, respectively. To discover the consistency, the node splitting analysis was performed to check the differences between direct and indirect comparisons among closed loops of each network. Sensitivity analysis was carried out to explore the robustness or consistency of the results and to determine whether a certain study has a high risk of bias. Egger’s test and Begg’s test were applied to determine publication bias across included trials where *p* values of <0.05 indicated publication bias. The statistical software R (R version 4.0.5, https://www.r-project.org/, accessed on 6 March 2022) and the R package GeMTC (version 1.0-1) were used to perform the NMA.

## 3. Results

### 3.1. Literature Search and Study Characteristics

**Figure 1:**
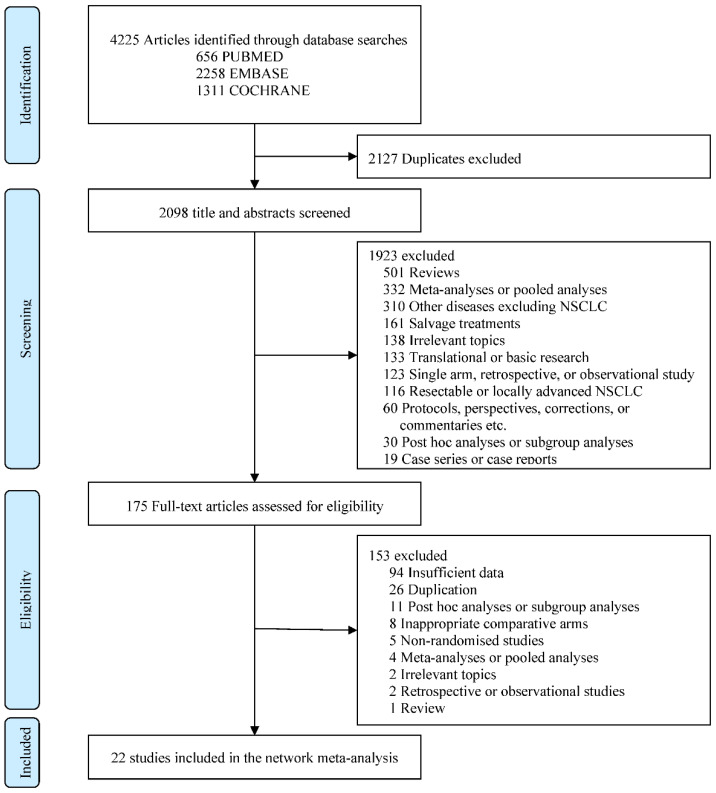
Preferred reporting items for systematic reviews and meta-analyses (PRISMA) flow diagram showing the selection process of studies included in Bayesian network meta-analysis.

A total of 4225 studies were retrieved during the literature search, from which 2127 duplicates were removed. Of the remaining studies, 1923 were excluded by inspecting titles and abstracts and then the full texts of 175 articles were reviewed. Finally, 22 randomized phase II or III trials were selected for the Bayesian NMA [13,15,16,17,19,20,21,22,23,24,25,26,27,28,29,30,31,32,33,34,41,42,43]. A flow diagram illustrating the process of literature selection is shown in Figure 1.

### 3.2. Characteristics of the Included Studies

The detailed characteristics of the included trials are summarized in Table 1. From 22 eligible studies, a total of 5237 NSCLC patients with high PD-L1 expression were included in this NMA. The patients received one of the following 19 treatment strategies: pembrolizumab, pembrolizumab plus doublet, atezolizumab, atezolizumab plus doublet, pembrolizumab plus ipilimumab, atezolizumab plus bevacizumab plus doublet, bevacizumab plus doublet, nivolumab, nivolumab plus ipilimumab, nivolumab plus ipilimumab plus doublet, nivolumab plus bevacizumab plus doublet, durvalumab, durvalumab plus tremelimumab, durvalumab plus tremelimumab plus doublet, camrelizumab plus doublet, tislelizumab plus doublet, sintilimab plus doublet, cemiplimab, and doublet chemotherapy.

### 3.3. Network Analysis Diagrams

**Figure 2:**
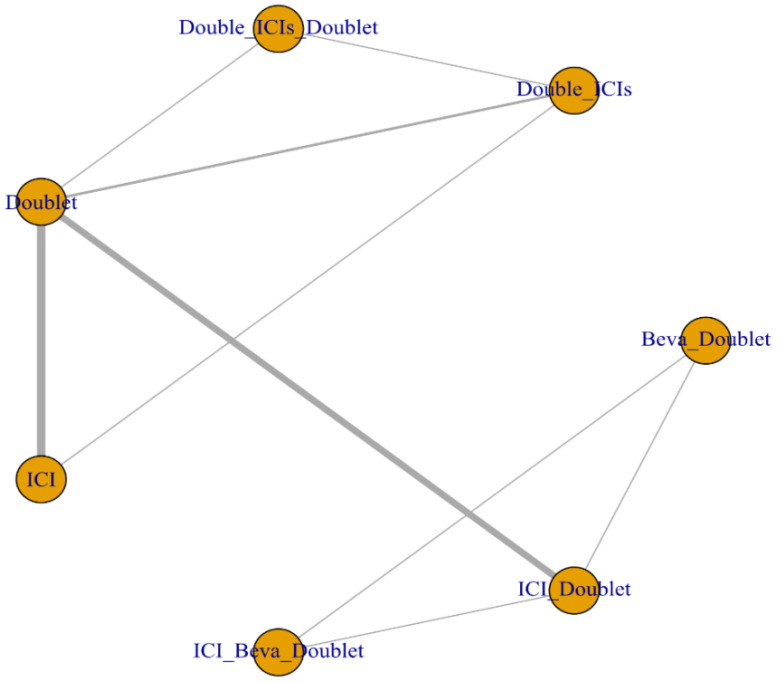
The network analysis diagram. Abbreviations: Beva: bevacizumab; ICI: immune checkpoint inhibitor; Doublet: doublet chemotherapy.

In this NMA, the treatment regimens were assigned into one of the following nodes: ICI monotherapy, ICI plus doublet, double ICIs, double ICIs plus doublet, ICI plus bevacizumab plus doublet, or doublet chemotherapy. The network analysis diagrams for OS and PFS are shown in Figure 2.

### 3.4. Risk of Bias Assessment

Since all studies were well-designed randomized controlled trials, the risk of bias was low in general across the studies (Figure S1). Although there was no information about the methods of randomization and allocation concealment in several trials, selection and attrition bias seemed to be minimal. However, the studies with open-labeled design (63.6%) were scored as having a high risk of bias in terms of blinding of participants and personnel. Because almost all studies were analyzed based on the intention-to-treat population and reported sufficient endpoints, a low risk of bias was observed with respect to the incomplete outcome data and selective reporting.

### 3.5. Progression-Free Survival

Seven network nodes covering 17 treatment regimens were included in the Bayesian NMA for PFS. The Gelman–Rubin diagnostic statistic value of 1.006 supported the model convergence, and the statistical heterogeneity was low across the trials (*I*^2^ = 14%) by fitting the random-effects model with appropriate informative prior distributions. Egger’s and Begg’s tests with a funnel plot indicated that there was no significant publication bias (Egger’s *p* = 0.300, Begg’s *p* = 0.082). The node-splitting model indicated that there were no significant differences between direct and indirect comparisons, suggesting no inconsistency in the network (Figure S2A). Sensitivity analysis showed that the results were relatively stable except for some small changes of SUCRA values.

**Figure 3:**
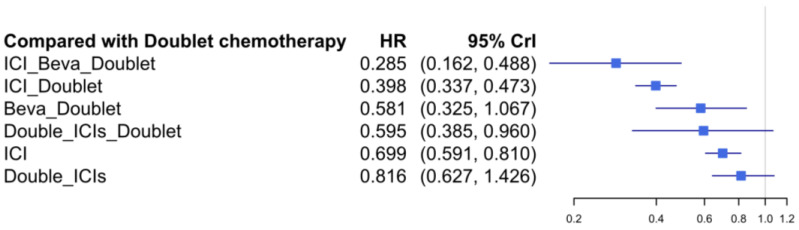
Network forest plot of treatment regimens compared with doublet chemotherapy for PFS.

The forest plot revealed that four network nodes had significant superiority to doublet chemotherapy (Figure 3). ICI plus doublet chemotherapy had a significantly better PFS over ICI monotherapy (HR = 0.571, 95% CrI: 0.454–0.709). The relative effects of all network node pairs on PFS are summarized in Table 2. Based on the SUCRA values, ICI plus bevacizumab plus doublet chemotherapy had the highest probability of being the most effective regimen (98.1%), followed by ICI plus doublet chemotherapy (82.9%).

### 3.6. Overall Survival

In this Bayesian NMA, 14 treatment regimens were available for OS analysis and assigned into seven network nodes. Model convergence was confirmed based on the Gelman–Rubin diagnostic statistic value of 1.009 and diagnostic plots. Statistical heterogeneity was found to be low across the included trials (*I*^2^ = 0%) after applying the random-effects model with appropriate informative prior distributions. Significant publication bias was not observed when Egger’s and Begg’s tests with a funnel plot were performed (Egger’s *p* = 0.868, Begg’s *p* = 0.371). The node-splitting analysis revealed that there were no significant differences between the direct and indirect estimates, indicating no inconsistency in the network (Figure S2B). The consistency of results was verified by sensitivity analysis.

**Figure 4:**
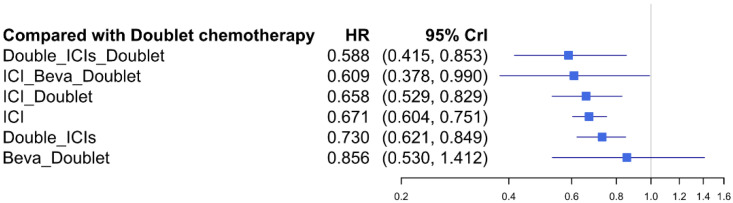
Network forest plot of each treatment strategy compared with doublet chemotherapy for OS.

Except for bevacizumab plus doublet chemotherapy, all treatments demonstrated a significantly reduced risk of death compared with doublet chemotherapy (Figure 4). However, none of the treatment regimens incorporating ICI showed significantly better OS than others in patients with NSCLC showing high PD-L1 expression. Especially, ICI plus doublet chemotherapy failed to show a significant superiority over ICI monotherapy (HR = 0.978, 95% CrI: 0.771–1.259).

The relative effects of all network node pairs for OS are presented in Table 2. The ranking of each treatment strategy was estimated according to the SUCRA values. Double ICIs plus doublet chemotherapy had the highest SUCRA value (79.1%), followed by ICI plus bevacizumab plus doublet chemotherapy (73.4%). ICI plus doublet chemotherapy (64.9%) and ICI monotherapy (61.8%) had a similar SUCRA value, indicating that they are equally effective against NSCLC with high PD-L1 expression in terms of OS.

### 3.7. Safety Analysis

Safety was analyzed according to all-grade adverse events (AEs) and Grade 3–5 AEs. Bayesian NMA included all network nodes. The incidence of toxicities was lowest for ICI monotherapy followed by double ICIs and doublet chemotherapy in both analyses (Table 3). Especially, ICI monotherapy and double ICIs showed significantly lower odds ratios (ORs) compared to the rest of the regimens combined with ICIs. The addition of chemotherapy and/or anti-angiogenic drug to ICIs elevated the toxicity.

**Figure 5:**
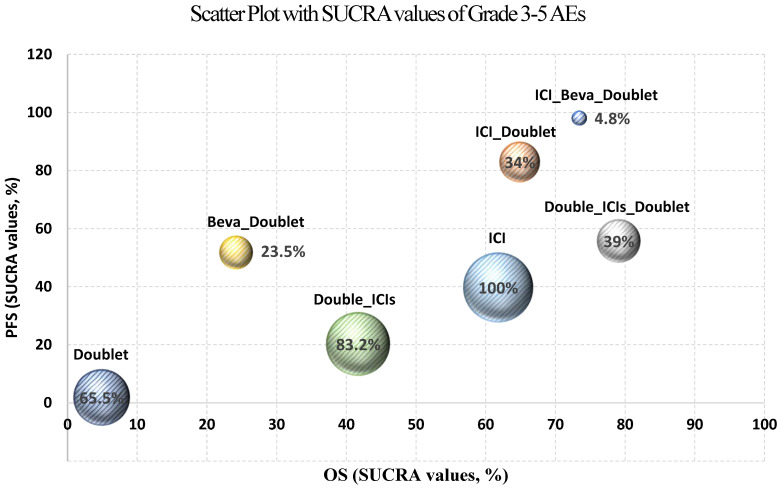
Scatter plot for efficacy and safety based on the SUCRA values (%). The size of each circle is weighted by the SUCRA values of Grade 3–5 AEs. Abbreviations: Beva: bevacizumab; ICI: immune checkpoint inhibitor; Doublet: doublet chemotherapy; SUCRA: surface under the cumulative ranking curve.

The Bayesian NMA results for the efficacy and safety were summarized in the scatter plot based on the SUCRA values of OS, PFS, and Grade 3–5 AEs in Figure 5.

## 4. Discussion

For this Bayesian NMA, we analyzed survival data of a total of 5237 patients from 22 randomized phase II or III trials in the first-line treatment setting for advanced NSCLC [13,15,16,17,19,20,21,22,23,24,25,26,27,28,29,30,31,32,33,34,41,42,43]. Our study used the most recent clinical outcomes and the most appropriate statistical methods for ICI immunotherapy-specific considerations. This NMA focused on patients whose tumors had high PD-L1 expression (≥50%). Based on the SUCRA values, ICI plus bevacizumab plus chemotherapy or ICI plus chemotherapy is likely to be the best option in terms of OS.

An increasing number of studies have suggested that there may be the synergistic anti-tumor effects between ICIs and chemotherapy. Cytotoxic agents may exhibit positive immuno-modulatory effects by releasing a high level of tumor antigens and changing the tumor micro-environment [44,45]. Accordingly, the combination of ICI and chemotherapy may reveal greater efficacy than chemotherapy alone, particularly in patients with lower PD-L1 expression levels. In fact, many randomized clinical trials have suggested that combining an anti-PD-1 mAb (pembrolizumab, camrelizumab, tislelizumab, sintilimab) or anti-PD-L1 inhibitor (atezolizumab) with platinum-doublet chemotherapy could significantly improve PFS or OS compared with chemotherapy alone in both squamous and nonsquamous advanced NSCLC, irrespective of the level of PD-L1 expression [16,18,19,23,32,34,41].

Besides ICIs targeting PD-l/PD-L1, cytotoxic T-lymphocyte-associated protein-4 (CTLA-4) checkpoint inhibitors also enhance T-cell activity against tumors with different complementary mechanisms. The first phase III study of dual immunotherapy, CheckMate 227, investigated the efficacy of nivolumab plus ipilimumab compared with platinum-based chemotherapy as frontline treatment of advanced NSCLC without *EGFR* or *ALK* mutations [25]. The updated results of the CheckMate 227 part 1 were recently reported [41]. With a median follow-up of 54.8 months, OS remained longer with nivolumab plus ipilimumab versus chemotherapy not only in patients with PD-L1 greater than or equal to 1% (HR = 0.76, 95% CI: 0.65–0.90) but also in patients with PD-L1 less than 1% (HR = 0.64, 95% CI: 0.51–0.81). In the CheckMate 9LA trial, first-line nivolumab plus ipilimumab combined with two cycles of chemotherapy improved OS versus chemotherapy alone (median OS 15.6 vs. 10.9 mo, HR = 0.66, 95% CI: 0.55–0.80) in patients with advanced NSCLC [26]. Interestingly, Ando et al. indirectly compared nivolumab plus ipilimumab versus other immunotherapies using NMA in PD-L1 positive (≥1%) advanced NSCLC [46]. The SUCRA ranking showed that pembrolizumab plus doublet chemotherapy had the highest efficacy for PFS, followed by nivolumab plus ipilimumab, nivolumab, doublet chemotherapy, and pembrolizumab. The safety outcome analysis revealed that nivolumab plus ipilimumab was well tolerated compared to existing immunotherapy regimens. These results indicate the possibility of dual immunotherapy with nivolumab and ipilimumab as a new therapeutic option in PD-L1-positive advanced NSCLC.

For patients with advanced NSCLC expressing PD-L1 of at least 50%, the results from several randomized studies indicate that ICI monotherapy is superior to chemotherapy in terms of both survival benefits and toxicity profile [14,24]. In the KEYNOTE-024 study, pembrolizumab provided meaningful survival benefits in both PFS (median 7.7 vs. 5.5 mo, HR = 0.50, 95% CI: 0.39–0.65) and OS (median 26.3 vs. 13.4 mo, HR = 0.62, 95% CI: 0.48–0.81) versus chemotherapy as first-line therapy for metastatic NSCLC with PD-L1 tumor proportion score greater than 50% [13]. In the EMPOWER-Lung 1 study, cemiplimab, a fully human, hinge-stabilized, immunoglobulin G4, anti-PD-1 mAb, also significantly improved PFS (median 8.2 vs. 5.7 mo, HR = 0.54, 95% CI: 0.43–0.68) and OS (median did not reach vs. 14.2 mo, HR = 0.57, 95% CI: 0.42–0.77), compared with chemotherapy in patients with advanced NSCLC expressing PD-L1 of at least 50% [24]. The Impower150 study was the first phase III trial to evaluate ICI (atezolizumab) in combination with an anti-angiogenic agent (bevacizumab) plus chemotherapy (paclitaxel and carboplatin) as frontline treatment of advanced nonsquamous NSCLC [17]. The results indicated that adding atezolizumab to chemotherapy plus bevacizumab significantly prolonged OS (HR = 0.80, 95% CI: 0.67–0.95), compared with chemotherapy plus bevacizumab. Interestingly, the exploratory analyses found that median OS was longer in the atezolizumab plus chemotherapy arm versus the bevacizumab plus chemotherapy arm (23.3 vs. 11.2 mo, HR = 0.59, 95% CI: 0.39–0.90) in the SP263-defined PD-L1-high subgroup (PD-L1 expressing tumor cells ≥ 50%). In addition, the improvement of OS was also observed in the atezolizumab–bevacizumab–chemotherapy arm versus the bevacizumab–chemotherapy arm (median 21.8 vs. 11.2 mo, HR = 0.62, 95% CI: 0.40–0.94) in the PD-L1-high subgroup [17]. In the recent phase III KEYNOTE-598 study, however, adding ipilimumab to pembrolizumab failed to improve efficacy and was associated with greater toxicity than pembrolizumab monotherapy as first-line treatment for metastatic NSCLC with PD-L1 TPS ≥ 50% and no targetable EGFR or ALK aberrations [43].

As we reviewed above, available data indicate that the addition of ICIs to chemotherapy with or without an anti-angiogenic agent increases survival benefits in advanced NSCLC, regardless of PD-L1 expression proportions [16,17,18,19,23,32,34]. Except for the KEYNOTE-598 study [43], however, no randomized clinical trials comparing the efficacy of ICIs as monotherapy versus combination with other treatment options are now available for patients with advanced NSCLC highly expressing PD-L1. Because this subgroup may achieve greater survival benefits from anti-PD-1/PD-L1 mAbs than chemotherapy, sparing those patients the risk of increased toxicities with the combination of other agents should be an important consideration.

In this Bayesian NMA of 22 randomized phase II or III trials with a total of 5237 patients, we indirectly compared survival outcomes and AEs of the seven treatment nodes (ICI monotherapy, ICI plus doublet chemotherapy, double ICIs with or without doublet chemotherapy, bevacizumab plus doublet chemotherapy with or without ICI, and doublet chemotherapy) as first-line treatment for advanced NSCLC with high PD-L1 expression. The toxicities were most tolerable for ICI monotherapy followed by double ICIs and doublet chemotherapy. Especially, ICI monotherapy and double ICIs showed significantly lower OR compared to the rest of the treatment regimens. The addition of chemotherapy and/or an anti-angiogenic drug to ICIs elevated the toxicities. Other network meta-analyses have also demonstrated that ICI monotherapy had significantly lower odds of any AEs than chemotherapy or a combination of ICI and chemotherapy [4,36]. In terms of PFS, four network nodes (ICI monotherapy, ICI plus chemotherapy, and bevacizumab plus chemotherapy with or without ICI) showed significant superiority, compared with chemotherapy alone. Interestingly, ICI plus chemotherapy had a significantly better PFS over ICI monotherapy (HR = 0.571, 95% CrI: 0.454–0.709). When the ranking of each treatment was estimated according to SUCRA values, ICI plus bevacizumab plus chemotherapy had the highest probability of being the most effective regimen (98.1%), followed by ICI plus chemotherapy (82.9%). In terms of OS, all treatment regimens except for bevacizumab plus chemotherapy demonstrated longer survival compared with chemotherapy alone. However, none of treatment regimens incorporating ICI showed significantly better OS than others. Especially, ICI plus chemotherapy failed to show a significant superiority over ICI monotherapy (HR = 0.978, 95% CrI: 0.771–1.259), indicating that ICI plus chemotherapy has no survival advantage compared with ICI monotherapy for patients with PD-L1 expression of at least 50%. Based on the SUCRA values, dual immunotherapy plus chemotherapy had the highest value (79.1%), followed by ICI plus bevacizumab plus chemotherapy (73.4%). However, it should be considered that only two studies were included in these treatment nodes. Moreover, dual immunotherapy (64.9%) and ICI monotherapy (61.8%) have similar SUCRA values, suggesting that they are equally effective in terms of OS against advanced NSCLC with high PD-L1 expression. Our findings were in concordance with the result from the recent meta-analysis of randomized controlled trials [36]. Li et al. compared the efficacy and safety of PD-1/PD-L1 inhibitors plus chemotherapy versus PD-1/PD-L1 inhibitors in advanced NSCLC using a network analysis. When stratifying patients according to PD-L1 expression level, patients with high PD-L1 expression receiving PD-L1 inhibitors plus chemotherapy had improved PFS, but not OS, compared to PD-L1 inhibitors as monotherapy.

Several limitations of this study need to be mentioned. First, this NMA was performed using aggregated data of results from the eligible trials, not individual patient data. Second, this study also included a randomized phase II study, and data from this kind of study may not be as reliable as data from phase III trials. However, only a single phase II study was included in the NMA, and thus it is less likely to have debatable impacts on the results [18]. Third, only one or two studies were included in two treatment nodes (double ICI plus doublet and ICI plus bevacizumab plus doublet), which could result in estimates with lower statistical power. Fourth, we did not stratify patients according to the histology (squamous or non-squamous) because of the limited number of available studies for each histology. Finally, the IHC methods measuring PD-L1 expression level were different among studies, which might cause patients to be misclassified.

In conclusion, we combined both direct and indirect evidence in this NMA of randomized trials to suggest frontline treatments for advanced NSCLC with high PD-L1 expression (≥50%). The results indicated that treatment regimens combined with immunotherapy reveal better survival outcomes compared with chemotherapy alone. Based on the SUCRA values, ICI plus bevacizumab plus chemotherapy or ICI plus chemotherapy might to be the best option in terms of OS. However, there was no significant difference in survival outcomes among treatment regimens combined with immunotherapy. Moreover, ICI plus chemotherapy failed to reveal significant survival benefits over ICI monotherapy. In addition, ICI monotherapy was most tolerable in terms of AEs, followed by double ICIs and doublet chemotherapy. In terms of both OS and safety, therefore, ICI monotherapy may also be reasonable as first-line treatment for advanced NSCLC with high PD-L1 expression and no targetable aberrations. Considering no prospective direct comparison is now available, however, the choice of treatment should be determined based on patient-specific factors after open discussion with the patient on the benefits, cost, and risks of each option. Randomized clinical trials are still warranted in order to identify the best therapeutic strategy for patients with advanced NSCLC highly expressing PD-L1.

## Figures and Tables

**Table 1:** Main characteristics of the 22 studies included in the Bayesian network meta-analysis.

Study [Ref]	Sample Size	Histology	* PD-L1 Status: n (%)	Intervention Arm	Control Arm	OS	PFS
KEYNOTE-024 [13]	305	NSCLC	≥50%: 305 (100)	Pembrolizumab	Doublet chemotherapy	0.62 (0.48–0.81)	0.50 (0.39–0.65)
KEYNOTE-042 [15]	1274	NSCLC	≥50%: 599 (47)	Pembrolizumab	Doublet chemotherapy	0.68 (0.57–0.82)	0.85 (0.72–1.02)
KEYNOTE-189 [29]	616	Nonsquamous	≥50%: 202 (33)	Pembrolizumab + Doublet chemotherapy	Doublet chemotherapy	0.59 (0.40–0.86)	0.35 (0.25–0.49)
KEYNOTE-407 [16]	559	Squamous	≥50%: 146 (26)	Pembrolizumab + Doublet chemotherapy	Doublet chemotherapy	0.79 (0.52–1.21)	0.37 (0.24–0.58)
KEYNOTE-598 [43]	568	NSCLC	≥50%: 568 (100)	Pembrolizumab + ipilimumab	Pembrolizumab	1.08 (0.85–1.37)	1.06 (0.86–1.30)
IMpower110 [21]	554	NSCLC	TC3 or IC3: 205 (37)	Atezolizumab	Doublet chemotherapy	0.59 (0.40–0.89)	0.63 (0.45–0.88)
IMpower130 [19]	724	Nonsquamous	TC3 or IC3: 134 (19)	Atezolizumab + Doublet chemotherapy	Doublet chemotherapy	0.84 (0.51–1.39)	0.51 (0.34–0.77)
IMpower131 [20]	1021	Squamous	TC3 or IC3: 154 (15)	Atezolizumab + Doublet chemotherapy	Doublet chemotherapy	0.48 (0.29–0.81)	0.41 (0.25–0.68)
IMpower132 [22]	578	Nonsquamous	TC3 or IC3: 45 (8)	Atezolizumab + Doublet chemotherapy	Doublet chemotherapy	0.73 (0.31–1.73)	0.46 (0.22–0.96)
IMpower150 [30]	1047	Nonsquamous	≥50%: 206 (24)	1. Atezolizumab + Bevacizumab + Doublet chemotherapy	Bevacizumab + Doublet chemotherapy	0.70 (0.46–1.08)	0.42 (0.28–0.63)
				2. Atezolizumab + Doublet chemotherapy		0.76 (0.49–1.17)	0.62 (0.3–0.89)
CheckMate 026 [17]	541	NSCLC	≥50%: 214 (40)	Nivolumab	Doublet chemotherapy	0.90 (0.63–1.29)	1.07 (0.77–1.49)
CheckMate 9LA [26]	719	NSCLC	≥50%: 174 (26)	Nivolumab + Ipilimumab + Doublet chemotherapy	Doublet chemotherapy	0.66 (0.44–0.99)	0.61 (0.42–0.89)
CheckMate 227 [41]	1189	NSCLC	≥50%: 611 (51)	1. Nivolumab + Ipilimumab 2. Nivolumab	Doublet chemotherapy	0.70 (0.55–0.90)	-
MYSTIC [27]	1118	NSCLC	≥50%: 333 (30)	1. Durvalumab + Tremelimumab	Doublet chemotherapy	0.77 (0.56–1.07)	1.05 (0.72–1.53)
				2. Durvalumab		0.76 (0.55–1.04)	0.87 (0.59–1.29)
CameL [23]	412	Nonsquamous	≥50%: 50 (24)	Camrelizumab + Doublet chemotherapy	Doublet chemotherapy	-	0.39 (0.14–0.99)
CCTG BR 34 [42]	301	NSCLC	≥50%: 57 (19)	Durvalumab + Tremelimumab + Doublet chemotherapy	Durvalumab + Tremelimumab	0.56 (0.27–1.17)	0.62 (0.32–1.19)
RATIONALE 304 [33]	334	Nonsquamous	≥50%: 110 (33)	Tislelizumab + Doublet chemotherapy	Doublet chemotherapy	-	0.308 (0.167–0.567)
RATIONALE 307 [32]	360	Squamous	≥50%: 125 (35	Tislelizumab + Doublet chemotherapy	Doublet chemotherapy	-	0.46 (0.31–0.70)
ORIENT-11 [31]	397	Nonsquamous	≥50%: 168 (42)	Sintilimab + Doublet chemotherapy	Doublet chemotherapy	-	0.310 (0.197–0.489)
ORIENT-12 [34]	357	Squamous	≥50%: 121 (34)	Sintilimab + Doublet chemotherapy	Doublet chemotherapy	-	0.458 (0.302–0.695)
EMPOWER-Lung 1 [24]	710	NSCLC	≥50%: 563 (79)	Cemiplimab	Doublet chemotherapy	0.57 (0.42–0.77)	0.54 (0.43–0.68)
TASUKI-52 [28]	550	Nonsquamous	≥50%: 147 (27)	Nivolumab + Bevacizumab + Doublet chemotherapy	Bevacizumab + Doublet chemotherapy	-	0.55 (0.36–0.83)

Abbreviations: Ref: reference; Beva: bevacizumab; ICI: immune checkpoint inhibitor. * This network meta-analysis focused on patients with PD-L1 ≥ 50% or TC3/IC3.

**Table 2:** The league table for the relative effects of all pairs of the network nodes and ranking for the probability of each network node to be the best for PFS and OS based on the SUCRA values.

PFS							
**0.285** **(0.163, 0.493)**	**0.398** **(0.336, 0.473)**	**0.581** **(0.399, 0.854)**	0.595 (0.327, 1.068)	**0.699** **(0.605, 0.815)**	0.816 (0.641, 1.078)	**SUCRA 1.8%** **Doublet**	
**0.347** **(0.188, 0.632)**	**0.487** **(0.352, 0.652)**	0.712 (0.465, 1.059)	0.725 (0.379, 1.367)	0.856 (0.665, 1.073)	**SUCRA 20.2%** **Double_ICIs**		**SUCRA 79.1%** **Double_ICIs_Doublet**
**0.407** **(0.229, 0.716)**	**0.571** **(0.454, 0.709)**	0.832 (0.561, 1.235)	0.850 (0.462, 1.546)	**SUCRA 39.7%** **ICI**		**SUCRA 73.4%** **ICI_Beva_Doublet**	0.970 (0.533, 1.748)
**0.478** **(0.339, 0.678)**	0.671 (0.382, 1.19)	0.977 (0.492, 2.002)	**SUCRA 51.7%** **Beva_Doublet**		**SUCRA 64.9%** **ICI_Doublet**	0.923 (0.61, 1.442)	0.892 (0.598, 1.385)
**0.489** **(0.249, 0.949)**	0.686 (0.45, 1.032)	**SUCRA 55.7%** **Double_ICIs_Doublet**		**SUCRA 61.8%** **ICI**	0.978 (0.771, 1.259)	0.907 (0.555, 1.502)	0.877 (0.61, 1.294)
0.714 (0.423, 1.203)	**SUCRA 82.9%** **ICI_Doublet**		**SUCRA 41.7%** **Double_ICIs**	0.920 (0.793, 1.089)	0.902 (0.691, 1.201)	0.840 (0.51, 1.381)	0.808 (0.564, 1.188)
**SUCRA 98.1%** **ICI_Beva_Doublet**		**SUCRA 24.2%** **Beva_Doublet**	0.849 (0.493, 1.427)	0.783 (0.466, 1.28)	0.768 (0.487, 1.172)	0.709 (0.455, 1.118)	0.694 (0.372, 1.245)
	**SUCRA 4.9%** **Doublet**	0.856 (0.53, 1.412)	**0.730** **(0.621, 0.849)**	**0.671** **(0.604, 0.751)**	**0.658** **(0.529, 0.829)**	**0.609** **(0.378, 0.99)**	**0.588** **(0.415, 0.853)**
**OS**							

Abbreviations: Beva: bevacizumab; ICI: immune checkpoint inhibitor; Doublet: doublet chemotherapy. Bold indicates statistically significant differences.

**Table 3:** The league table presenting the ORs for all pairs of the network nodes and ranking for the probability of each network node to be the best for AE of Grade 3–5 and AE of all grades based on the SUCRA values.

AE of Grade 3–5							
**0.156** **(0.092, 0.265)**	**0.32** **(0.181, 0.558)**	**0.48** **(0.294, 0.783)**	0.669 (0.351, 1.246)	0.715 (0.452, 1.126)	0.816 (0.572, 1.167)	**ICI_Beva_Doublet** **SUCRA 4.8%**	
**0.191** **(0.112, 0.321)**	**0.391** **(0.222, 0.672)**	**0.588** **(0.36, 0.951)**	0.817 (0.429, 1.531)	0.877 (0.552, 1.374)	**Beva_Doublet** **SUCRA 23.5%**		**ICI** **SUCRA 99.7%**
**0.218** **(0.167, 0.284)**	**0.448** **(0.322, 0.613)**	**0.672** **(0.564, 0.8)**	0.932 (0.598, 1.439)	**ICI_Doublet** **SUCRA 34.0%**		**Double_ICIs** **SUCRA 83.0%**	**0.544** **(0.313, 0.919)**
**0.234** **(0.152, 0.363)**	**0.48** **(0.312, 0.732)**	0.72 (0.484, 1.084)	**Double_ICIs_Doublet SUCRA 39.0%**		**Doublet** **SUCRA 62.40%**	**0.465** **(0.286, 0.797)**	**0.253** **(0.18, 0.372)**
**0.325** **(0.266, 0.397)**	**0.667** **(0.508, 0.862)**	**Doublet** **SUCRA 65.5%**		**Double_ICIs_Doublet SUCRA 46.0%**	0.676 (0.285, 1.735)	**0.317** **(0.12, 0.918)**	**0.171** **(0.068, 0.471)**
**0.487** **(0.369, 0.652)**	**Double_ICIs** **SUCRA 83.2%**		**ICI_Doublet** **SUCRA 28.5%**	0.637 (0.218, 1.755)	**0.432** **(0.251, 0.728)**	**0.202** **(0.096, 0.423)**	**0.11** **(0.058, 0.208)**
**ICI** **SUCRA 100%**		**ICI_Beva_Doublet** **SUCRA 27.3%**	0.865 (0.256, 2.972)	0.547 (0.109, 2.719)	0.37 (0.098, 1.435)	**0.173** **(0.042, 0.755)**	**0.094** **(0.024, 0.382)**
	**Beva_Doublet** **SUCRA 3.5%**	0.414 (0.108, 1.386)	0.354 (0.085, 1.403)	0.223 (0.037, 1.208)	**0.152** **(0.033, 0.663)**	**0.071** **(0.014, 0.345)**	**0.039** **(0.008, 0.177)**
**AE of All Grades**							

Abbreviations: Beva: bevacizumab; ICI: immune checkpoint inhibitor; Doublet: doublet chemotherapy. Bold indicates statistically significant differences.

## Supplementary Materials

The following supporting information can be downloaded at: https://www.mdpi.com/article/10.3390/jcm11061492/s1. Table S1: Detailed characteristics of the 22 studies included in the Bayesian network meta-analysis. Figure S1: Graphic (A) and tabular representation (B) of risk of bias in all studies included. Figure S2: Node splitting analysis for PFS (A) and OS (B).
