# Supplementary material for: A Bayesian Network Meta-Analysis of First-Line Treatments for Non-Small Cell Lung Cancer with High Programmed Death Ligand-1 Expression
Source: J Clin Med. 2022 Mar 9;11(6):1492. doi: 10.3390/jcm11061492 (PMC8950568; doi:10.3390/jcm11061492)
Supplement: Supplementary file 1 [file jcm-11-01492-s001.zip › jcm-1552921-supplementary.pdf]

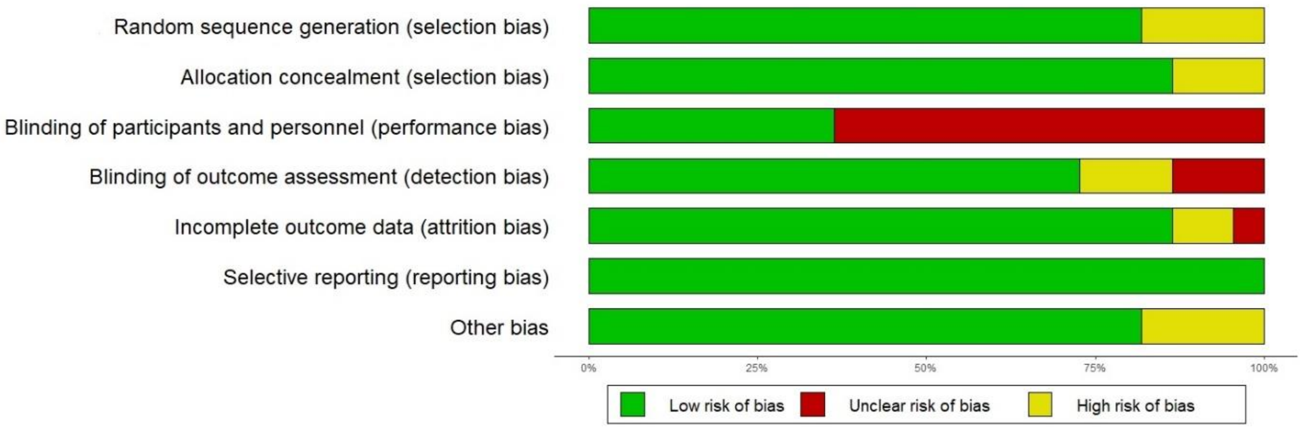


(**A**)

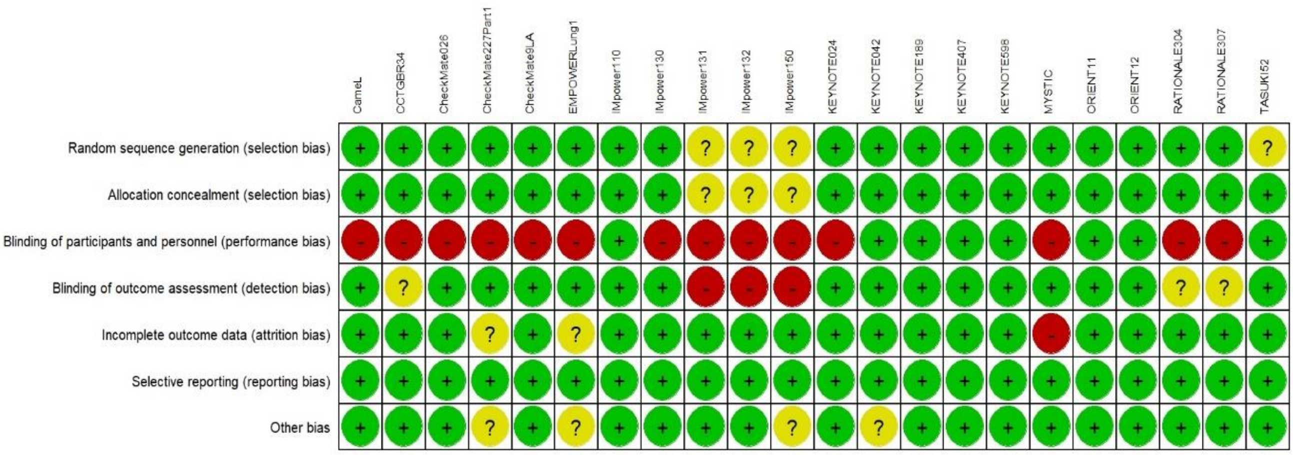


(**B**)

**Figure S1.** Graphic (**A**) and tabular representation (**B**) of risk of bias in all studies included.

(**A**)

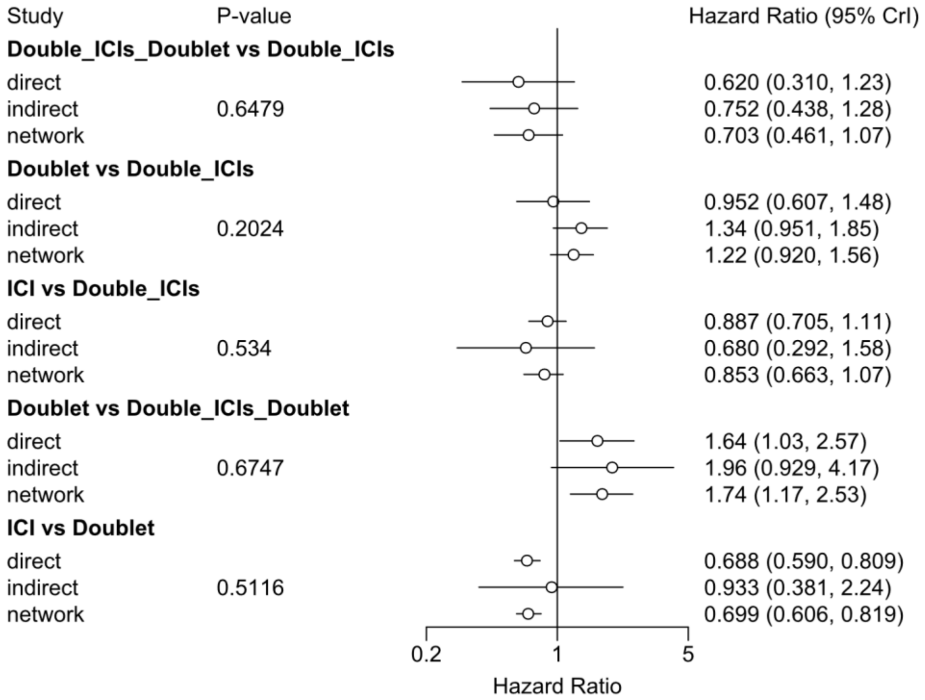


(**B**)

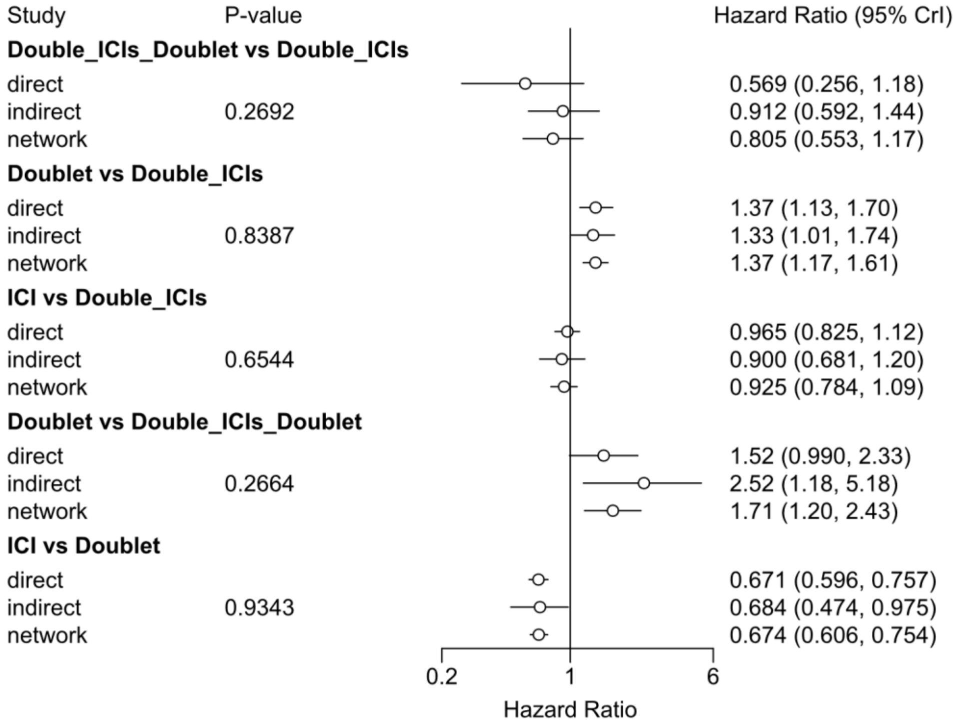


**Figure S2.** Node splitting analysis for PFS (**A**) and OS (**B**).

**Table S1.** Detailed characteristics of the 22 studies included in the Bayesian network meta-analysis.

| Study [ref] | Trial Design | Mutations | PD-L1 Criteria | PD-L1 Detection | Intervention Arm | Control Arm |
|---|---|---|---|---|---|---|
| **KEYNOTE-024** [13] | Phase III Open-label | Without EGFR or ALK alterations | ≥ 50 % | 22C3 pharmDx | Pembrolizumab 200mg Q3W | Pemetrexed 500mg/$m^2$ + Carboplatin AUC5~6 or Cisplatin 75mg/$m^2$ Q3W<br>Gemcitabine 1250mg/$m^2$ D1,8 + Carboplatin AUC5~6 or Cisplatin 75mg/$m^2$ Q3W<br>Paclitaxel 200mg/$m^2$ + Carboplatin AUC5~6 Q3W |
| **KEYNOTE-042** [15] | Phase III Open-label | Without EGFR or ALK alterations | ≥ 1 % | 22C3 pharmDx | Pembrolizumab 200mg Q3W | Pemetrexed 500mg/$m^2$ + Carboplatin AUC5~6 Q3W<br>Paclitaxel 200mg/$m^2$ + Carboplatin AUC5~6 Q3W |
| **KEYNOTE-189** [29] | Phase III Double-blind | Without EGFR or ALK alterations | All comers | 22C3 pharmDx | Pembrolizumab 200mg Q3W + Pemetrexed 500mg/$m^2$ + Carboplatin AUC5 or Cisplatin 75mg/$m^2$ Q3W | Pemetrexed 500mg/$m^2$ + Carboplatin AUC5 or Cisplatin 75mg/$m^2$ Q3W |
| **KEYNOTE-407** [16] | Phase III Double-blind | Unselected | All comers | 22C3 pharmDx | Pembrolizumab 200mg Q3W + Paclitaxel 200mg/$m^2$ + Carboplatin AUC6 Q3W | Paclitaxel 200mg/$m^2$ + Carboplatin AUC6 Q3W |
| **KEYNOTE-598** [43] | Phase III Double-blind | Without EGFR or ALK alterations | ≥ 50 % | 22C3 pharmDx | Pembrolizumab 200mg Q3W + ipilimumab 1mg/kg Q6W | Pembrolizumab 200mg Q3W |
| **IMpower110** [21] | Phase III Open-label | Without EGFR or ALK alterations | ≥ 1 % | SP142 | Atezolizumab 1200mg Q3W | Pemetrexed 500mg/$m^2$ + Carboplatin AUC6 or Cisplatin 75mg/$m^2$ Q3W<br>Gemcitabine 1000mg/$m^2$ D1,8 + Carboplatin AUC5 Q3W<br>Gemcitabine 1250mg/$m^2$ D1,8 + Cisplatin 75mg/$m^2$ Q3W |
| **IMpower130** [19] | Phase III Open-label | Without EGFR or ALK alterations | All comers | SP142 | Atezolizumab 1200mg + nab-Paclitaxel 100mg/$m^2$ + Carboplatin AUC6 Q3W | nab-Paclitaxel 100mg/$m^2$ + Carboplatin AUC6 Q3W |
| **IMpower131** [20] | Phase III Open-label | Unselected | All comers | SP142 | Atezolizumab 1200mg + nab-Paclitaxel 100mg/$m^2$ + Carboplatin AUC6 Q3W | nab-Paclitaxel 100mg/$m^2$ + Carboplatin AUC6 Q3W |
| **IMpower132** [22] | Phase III Open-label | Without EGFR or ALK alterations | All comers | SP142 | Atezolizumab 1200mg + Pemetrexed 500mg/$m^2$ + Carboplatin AUC6 or Cisplatin 75mg/$m^2$ Q3W | Pemetrexed 500mg/$m^2$ + Carboplatin AUC6 or Cisplatin 75mg/$m^2$ Q3W |
| **IMpower150** [30] | Phase III Open-label | Without EGFR or ALK alterations | All comers | SP142 | 1. Atezolizumab 1200mg + Bevacizumab 15mg/kg + Paclitaxel 200mg/$m^2$ + Carboplatin AUC6 Q3W<br>2. Atezolizumab 1200mg + Paclitaxel 200mg/$m^2$ + Carboplatin AUC6 Q3W | Bevacizumab 15mg/kg + Paclitaxel 200mg/$m^2$ + Carboplatin AUC6 Q3W |
| **CheckMate 026** [17] | Phase III Open-label | Without EGFR or ALK alterations | ≥ 1 % | 28-8 PharmDx | Nivolumab 3mg/kg Q2W | Gemcitabine 1250mg/$m^2$ D1,8 + Cisplatin 75mg/$m^2$ Q3W |

| | | | | | | |
|---|---|---|---|---|---|---|
| | | | | | | Gemcitabine 1000mg/m$^2$ D1,8 + Carboplatin AUC5 Q3W<br>Paclitaxel 200mg/m$^2$ + Carboplatin AUC6 Q3W<br>Pemetrexed 500mg/m$^2$ + Carboplatin AUC6 or Cisplatin 75mg/m$^2$ Q3W |
| **CheckMate 9LA** [26] | Phase III Open-label | Without EGFR or ALK alterations | All comers | 28-8 PharmDx | Nivolumab 350mg Q3W + Ipilimumab 1mg/kg Q6W + Pemetrexed 500mg/m$^2$ + Carboplatin AUC6 or Cisplatin 75mg/m$^2$ Q3W | Pemetrexed 500mg/m$^2$ + Carboplatin AUC6 or Cisplatin 75mg/m$^2$ Q3W |
| **CheckMate 227** [25,41] | Phase III Open-label | Without EGFR or ALK alterations | All comers | 28-8 PharmDx | 1. Nivolumab 3mg/kg Q2W + Ipilimumab 1mg/kg Q6W<br>2. Nivolumab 240mg Q2W | Pemetrexed 500mg/m$^2$ + Carboplatin AUC6 or Cisplatin 75mg/m$^2$ Q3W<br>Gemcitabine 1250mg/m$^2$ D1,8 + Cisplatin 75mg/m$^2$ Q3W<br>Gemcitabine 1000mg/m$^2$ D1,8 + Carboplatin AUC5 Q3W |
| **MYSTIC** [27] | Phase III Open-label | Without EGFR or ALK alterations | All comers | SP263 | 1. Durvalumab 20mg/kg + Tremelimumab 1mg/kg Q4W<br>2. Durvalumab 20mg/kg Q4W | Pemetrexed 500mg/m$^2$ + Carboplatin AUC6 or Cisplatin 75mg/m$^2$ Q3W |
| **CameL** [23] | Phase III Open-label | Without EGFR or ALK alterations | All comers | 22C3 pharmDx | Camrelizumab 200mg + Pemetrexed 500mg/m$^2$ + Carboplatin AUC5 Q3W | Pemetrexed 500mg/m$^2$ + Carboplatin AUC5 Q3W |
| **CCTG BR 34** [42] | Phase II Open-label | Without EGFR or ALK alterations | All comers | SP263 | Durvalumab 1500mg Q4W + Tremelimumab 75mg Q4W + Pemetrexed 500mg/m$^2$ + Carboplatin AUC6 or Cisplatin 75mg/m$^2$ Q3W | Durvalumab 1500mg + Tremelimumab 75mg Q4W |
| **RATIONALE 304** [33] | Phase III Open-label | Without EGFR or ALK alterations | All comers | SP263 | Tislelizumab 200mg + Pemetrexed 500mg/m$^2$ + Carboplatin AUC5 or Cisplatin 75mg/m$^2$ Q3W | Pemetrexed 500mg/m$^2$ + Carboplatin AUC5 or Cisplatin 75mg/m$^2$ Q3W |
| **RATIONALE 307** [32] | Phase III Open-label | Without EGFR or ALK alterations | All comers | SP263 | Tislelizumab 200mg + Paclitaxel 200mg/m$^2$ + Carboplatin AUC5 Q3W<br>Tislelizumab 200mg + nab-Paclitaxel 100mg/m$^2$ + Carboplatin AUC5 Q3W | Paclitaxel 200mg/m$^2$ + Carboplatin AUC5 Q3W |
| **ORIENT-11** [31] | Phase III Double-blind | Without EGFR or ALK alterations | All comers | 22C3 pharmDx | Sintilimab 200mg + Paclitaxel 200mg/m$^2$ + Carboplatin AUC5 or Cisplatin 75mg/m$^2$ Q3W | Paclitaxel 200mg/m$^2$ + Carboplatin AUC5 or Cisplatin 75mg/m$^2$ Q3W |
| **ORIENT-12** [34] | Phase III Double-blind | Without EGFR or ALK alterations | All comers | 22C3 pharmDx | Sintilimab 200mg + Gemcitabine 1000mg/m$^2$ D1,8 + Carboplatin AUC5 or Cisplatin 75mg/m$^2$ Q3W | Gemcitabine 1000mg/m$^2$ D1,8 + Carboplatin AUC5 or Cisplatin 75mg/m$^2$ Q3W |
| **EMPOWER-Lung 1** [24] | Phase III Open-label | Without EGFR or ALK alterations | ≥ 50 % | 22C3 pharmDx | Cemiplimab 350mg Q3W | Pemetrexed 500mg/m$^2$ + Carboplatin AUC5~6 or Cisplatin 75mg/m$^2$ Q3W<br>+ Paclitaxel 200mg/m$^2$ + Carboplatin AUC5~6 or Cisplatin 75mg/m$^2$ Q3W<br>Gemcitabine 1250mg/m$^2$ D1,8 + Carboplatin AUC5~6 or Cisplatin 100mg/m$^2$ Q3W |

| | | | | | | |
|---|---|---|---|---|---|---|
| **TASUKI-52** [28] | Phase III Double-blind | Without EGFR or ALK alterations | All comers | 28-8 PharmDx | Nivolumab 360mg + Bevacizumab 15mg/kg + Paclitaxel 200mg/m$^2$ + Carboplatin AUC6 Q3W | Bevacizumab 15mg/kg + Paclitaxel 200mg/m$^2$ + Carboplatin AUC6 Q3W |
